# The Anti-inflammatory Effects of Dietary Anthocyanins against Ulcerative Colitis

**DOI:** 10.3390/ijms20102588

**Published:** 2019-05-27

**Authors:** Shiyu Li, Binning Wu, Wenyi Fu, Lavanya Reddivari

**Affiliations:** 1 Department of Food Science, Purdue University, 745 Agriculture Mall Drive, West Lafayette, IN 47907, USA; li3291@purdue.edu (S.L.); wu1515@purdue.edu (B.W.); fu205@purdue.edu (W.F.); 2 Department of Plant Science, Penn State University, University Park, PA 16802, USA

**Keywords:** anthocyanins, anti-inflammatory, colitis, colonic inflammation

## Abstract

Ulcerative colitis (UC), which is a major form of inflammatory bowel disease (IBD), is a chronic relapsing disorder of the gastrointestinal tract affecting millions of people worldwide. Alternative natural therapies, including dietary changes, are being investigated to manage or treat UC since current treatment options have serious negative side effects. There is growing evidence from animal studies and human clinical trials that diets rich in anthocyanins, which are pigments in fruits and vegetables, protect against inflammation and increased gut permeability as well as improve colon health through their ability to alter bacterial metabolism and the microbial milieu within the intestines. In this review, the structure and bioactivity of anthocyanins, the role of inflammation and gut bacterial dysbiosis in UC pathogenesis, and their regulation by the dietary anthocyanins are discussed, which suggests the feasibility of dietary strategies for UC mitigation.

## 1. Anthocyanins

Anthocyanins, which is a clan of flavonoids, are water-soluble polyphenolic pigments that are responsible for the pigmentation of anthocyanin-rich foods including fruits (black plums, blackberries, blueberries, and grapes), vegetables (black plums, blackberries, blueberries, and grapes), and grains (black rice, red rice, and black soybeans) [1,2,3,4,5]. Different crops vary in the composition and the content of anthocyanins ranging from 0.1% to 1.0% [6,7]. Additionally, oxidation, enzymolysis, and environmental factors such as temperature, light, and pH can alter anthocyanin levels [8]. Previous studies showed that malonylation enhanced the stability of anthocyanins in water [9]. Most of the anthocyanins exert better stability under acidic conditions while high pH leads to anthocyanin degradation [10,11]. pH-dependent reversible structure transformation occurs between the following forms: flavylium cation (red), quinonoidal base (blue), carbinol pseudobase (colorless), and chalcone (colorless) [12] in aqueous solution [13]. In plants, anthocyanins aid in pollination and anthocyanin pigments can serve as natural food colorants [11,14].

**Figure 1:**
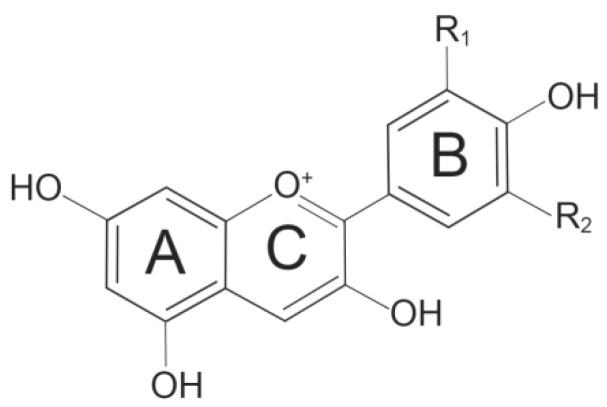
The basic structure of anthocyanin.

**Figure 2:**
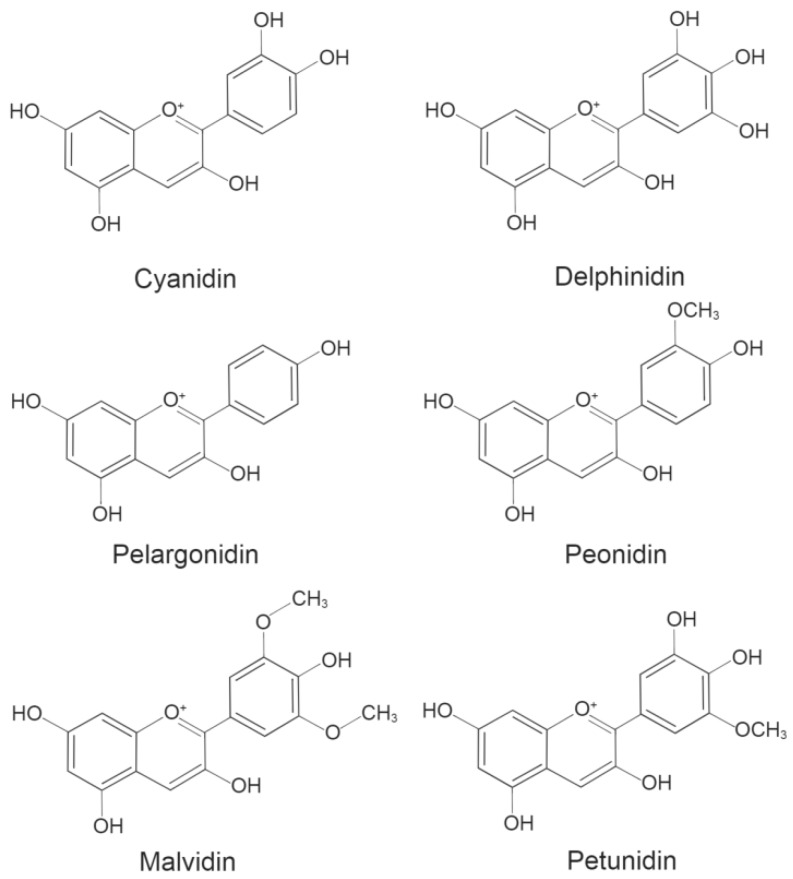
Structures of six major anthocyanidins.

Anthocyanins are naturally present in plants as glycosides carrying glucose, galactose, arabinose, rhamnose, and xylose [15]. Deglycosylated anthocyanins known as anthocyanidins are unstable and rarely found in nature [16]. The instability of anthocyanidins is due to the presence of flavylium ion and its peculiar electron distribution [17]. To date, a total of 27 aglycones and over 700 anthocyanins have been identified based on their chemical structures [1,18]. Anthocyanins share a basic C-6 (A ring)-C-3 (C ring)-C-6 (B ring) carbon skeleton (Figure 1) with a varying number of hydroxyl groups and sugars with different degrees of methylation [19]. Approximately 665 natural anthocyanins are derived from six commonly found anthocyanidins (Figure 2): cyanidin (Cy), peonidin (Pn), pelargonidin (Pg), malvidin (Mv), delphinidin (Dp), and petunidin (Pt) [13,20]. 

Red-colored or blue-colored fruits, vegetables, and grains serve as sources of various anthocyanins. For example, 100 g kokum can provide 1000 to 2400 mg anthocyanins [21], 100 g strawberry contains 13-315 mg anthocyanins [22], and 100 g red wine grapes supply 30-750 mg anthocyanins [23]. As reported by Raul Zamora-Ros et al., daily consumption of anthocyanins varies depending on the region, weather condition, gender, and lifestyle [24]. Among all European regions that are investigated, Italy had the highest daily anthocyanin intake (~43.74 mg/day), with men consuming 49% more anthocyanins daily than women. The opposite pattern was observed in the UK, where daily anthocyanin intake of women is 21% higher than men [24]. The estimated anthocyanin daily intake in the US is about 11.6 mg/day [25]. 

### 1.1. Anthocyanin Bioavailability

The structure of anthocyanins is a key factor that determines their bioavailability and bioactivity. Bioavailability is defined as the rate and extent to which a compound is absorbed and utilized by the organism to perform multiple physiological effects [26]. Thus, the bioavailability has been considered as an essential index in evaluating the efficacy of bioactive compounds. Absorption is the main factor that influences the bioavailability of anthocyanins. The absorption rate varies depending on the molecular size, sugar moiety, and acylated groups. Moreover, the interference by other materials within the food matrix is also a considerable factor that affects the absorption. An *in vitro* study conducted by Yi et al. showed that anthocyanins with more free hydroxyl groups and fewer OCH_3_ groups had lower bioavailability [27]. Anthocyanidin-glucosides exhibited higher bioavailability than anthocyanidin-galactosides, while non-acylated anthocyanins have better absorption than the acylated ones [28,29]. Studies also found that anthocyanins can be absorbed mainly in their intact glycosidic forms through the stomach and small intestine [19]. Anthocyanins were detected in the plasma within a few minutes after intake, which indicates the rapid absorption in the stomach [30]. Talavera et al. indicated that 19% to 37% of bilberry anthocyanins were absorbed by gastric fluid within 30 min [31]. An *in vivo* study showed that the highest absorption of anthocyanins occurred in the jejunum (55.3 ± 7.6%) whereas minor absorption occurred in the duodenum (10.4 ± 7.6%), which supports the role of the small intestine as a major site for anthocyanin absorption [32]. Unabsorbed anthocyanins travel down to the colon. However, both humans [33] and mice studies [34] demonstrated that most of the cyanidin-3-glucosides (C3G) that enter the large intestine was excreted in feces. Although anthocyanins display high absorption in the gastrointestinal tract, the bioavailability of anthocyanins is less than 1% [35,36,37]. Recent studies suggest that anthocyanins similar to other flavonoids are metabolized by colonic microbiota (Table 1) [38,39] and the metabolic function might be a direct result of metabolomic indicators rather than the bioavailability [40].

### 1.2. Anthocyanin and Human Health

Anthocyanins have been indicated to be a group of bioactive compounds with numerous health benefits because of their anti-inflammatory, anti-oxidant, anti-obesity, anti-angiogenesis, anti-cancer, anti-diabetes, anti-microbial, neuroprotection, and immunomodulation properties (Table 2) [9]. Studies demonstrated that anthocyanins exhibited a strong attenuating effect against colitis [46] and colon cancer [47]. The anti-angiogenic effect of anthocyanins has been proven on human esophageal and intestinal microvascular endothelial cells [48]. Significant evidence supports the preventive efficacy of anthocyanins against many neurodegenerative diseases such as Parkinson’s disease and Alzheimer’s disease [49]. Previous studies indicated that middle-aged and older-aged women with a high consumption of anthocyanin-rich foods exhibited 32% and 18% reduction in risk of myocardial infarction, respectively [50,51]. Additionally, human obesity prevention and blood glucose tolerance effects of anthocyanin have also been reported [52,53]. Anthocyanins have been shown to reduce oxidative stress either by scavenging reactive oxygen species or by inducing anti-oxidant enzymes. Anthocyanins in black currant skin induced the anti-oxidant enzymes and eased the oxidative stress through activation of the Nrf2 signaling pathway [54]. Moreover, oxidative stress can increase inflammation by enhanced pro-inflammatory gene expression and inflammation, which, in turn, can lead to oxidative stress (ref-curcumin review). Antioxidative effects of anthocyanins can contribute to the anti-inflammatory properties, but we will not be covering the anti-oxidative effects of anthocyanins. In this review, we will focus on the anti-inflammatory effects of anthocyanins against ulcerative colitis (UC). 

## 2. Ulcerative Colitis Pathogenesis

Ulcerative colitis (UC), which is a chronic and idiopathic inflammatory disease of the colon, is one of the major forms of inflammatory bowel disease (IBD). UC occurs with several clinical symptoms, such as abdominal and/or rectal pain, diarrhea, bloody stool, weight loss, fever, and even rectal prolapse under the severe scenario. UC is also associated with an increased risk of colon cancer [78]. Recent studies have identified various genetic and environmental factors involved in UC pathogenesis. Studies showed that UC is more common in western and northern countries when compared with eastern countries [79]. The peak age for UC occurrence is 30 to 40 years [80] and people with infection history of nontyphoid *Salmonella* or *Campylobacter* exhibit eight to 10 times more risk to develop UC in later years [81]. Moreover, former smoking [82], high fat, and/or sugar diets [83], hormone replacement, and anti-inflammatory therapy have been shown to be closely related to increased risk of UC [83,84,85,86]. Collectively, UC is a wide-spread inflammatory disease all over the world and can worsen the quality of a patient’s life due to the continuous, serious clinical symptoms, possible complications, and sustained medical intervention [46].

### 2.1. Impaired Barrier Function and Inflammatory Signaling Pathways 

Pathologically, UC is characterized by epithelial ulceration, immune cell infiltration in the lamina propria, crypt abscess, enlarged spleen and liver, and impaired intestinal epithelial barrier function [87,88]. The integrity of the mucus layer, the production, and assembly of tight junction (TJ) proteins are two main factors to evaluate intestinal barrier function. Decreased thickness of the mucus layer and expression of TJ proteins (claudins, occludin, and zonula occluden-1 (ZO-1)) and increased gut permeability against bacterial product have been found in chemical-induced colitis models [89,90,91]. Weakened epithelium barrier function with increased permeability allows for the translocation of commensal bacteria and microbial products into the bowel wall and, ultimately, activates the innate and adaptive immune response. 

Several components involved in the gut immunity have been highly implicated in UC pathogenesis including dendritic cells (DCs), macrophages, eosinophils, neutrophils, T-cells, B-cells, and their secreted cytokines and chemokines. Disturbed responses of effector T-cells, T-helper 2 (Th2), and Th17 were observed in the context of UC. Th2 produces cytokines such as tumor necrosis factor alpha (TNF-α), IL-5, IL-6, and IL-13 while Th17 produces IL-17A, IL-21, and IL-22 to activate multiple target cells and downstream signaling pathways to exert their pro-inflammatory functions by binding to corresponding receptors [92,93,94]. TNF, IL-6, IL-17A, and IL-22 levels are significantly elevated in experimental colitis and UC patients [95,96,97]. TNF binds to TNFR1 and TNFR2, followed by the recruitment of TNF receptor-associated factor 2 (TRAF2) and activation of JNK-dependent kinase cascade, MEKK kinase cascade, and the nuclear factor-κB (NF-κB) signaling pathway to induce apoptosis, necroptosis, and production of other pro-inflammatory cytokines [93,98]. IL-6, which is another key cytokine in UC, functions in governing the proliferation and survival of Th1 and Th2 cells by pairing with IL-1β to serve as a signaling molecule for the generation of regulatory B cells and mediate STAT3-dependent T cell production of anti-inflammatory cytokine IL-10 [99,100]. IL-13 is identified to be an important effector cytokine in UC to induce epithelial cell apoptosis and compromise epithelial restitution velocity [101]. Similar to IL-10, IL-22 is an anti-inflammatory cytokine involved in wound healing and production of defensins and mucins against bacterial invasion [102]. Up-regulation of antigen-presenting cells (APCs) expressing Toll-like receptors 4 (TLR4) is another scenario in human UC. Binding of TLR4 to ligand lipopolysaccharide (LPS) triggers activation of NF-κB via protein adaptor MyD88 and allows for transcription of numerous inflammatory genes such as TNF-α, IL-6, IL-1β, and cyclooxygenase-2 (COX-2) [103,104].

### 2.2. Gut Microbiota Dysbiosis

Gut-commensal bacteria have a profound impact on host health and the pathogenesis of UC. Gut microbiota play an important role in nutrition, immunomodulation, and various metabolic processes to exhibit their beneficial function in maintaining gut homeostasis [105]. Intestinal symbiotic bacteria help in maintaining intestinal stability and prevent the colonization of pathogens. For example, capsular polysaccharide A (PSA) of *Bacteroides fragilis* can be delivered to regulatory T cells (Tregs) to induce interleukin-10 (IL-10) production against experimental colitis [106]. Gut microbial metabolites such as short-chain fatty acids (SCFAs) produced via dietary fiber fermentation also play a key role in maintaining colon health [107,108]. Moreover, utilization of non-pathogenic commensal bacteria *Lactobacillus* and *Bifidobacterium* as probiotics have shown promising results in UC remission [109,110,111]. Dysbiosis of gut bacteria with respect to diversity and bacterial load might be one of the contributing factors to the pathogenesis of UC because of the overstimulation of mucosal immune response [112]. 16S rRNA sequencing performed on fecal and biopsy samples from UC patients revealed a reduction in bacterial alpha diversity and an increase in total bacterial load compared to healthy subjects [113]. Evident reductions of bacterial phyla in UC patients include *Bacteroidetes* and *Firmicutes*, among which two SCFA producing bacteria from the genus, *Phascolarctobacterium*, and *Roseburia*, were significantly reduced in abundance [114]. Conversely, concentrations of adhesive invasive *E.coli* have increased under the UC condition [115]. The impaired intestinal mucosal barrier in predisposed subjects is marked as one of the early events of UC as the consequence of gut microbial dysbiosis. Gut bacterial dysbiosis-induced release of enterotoxins lead to increased intestinal permeability and immune dysfunction [116,117].

## 3. Anthocyanin and Ulcerative Colitis

The rapidly rising incidence of UC makes the prevention, therapy, and control of this disease important. Current standard UC therapies utilize aminosalicylates, immunosuppressants, and biologicals to interfere with the inflammatory cascade. However, the long-term use of these therapeutic agents may result in undesirable side effects such as vomiting, nausea, headache, and fatigue [91]. Hence, there is an urgent demand for developing effective and evidence-based therapeutic strategies with minimal side effects. Bioactive compounds such as anthocyanins might be potential candidates against UC [92]. There is extensive evidence from laboratory animal studies and human clinical trials that dietary anthocyanins derived from fruits and vegetables protect against intestinal inflammation and provide health benefits to the colon [48,118,119,120]. Anthocyanins exert its anti-inflammatory effects against UC through effective protection of intestinal mucosal integrity, restoration of epithelial barrier function, immunomodulation, and regulation of gut microbiota [90,121].

### 3.1. Anthocyanins: Mucosal Integrity and Intestinal Epithelial Barrier Function

The integrity of the mucus layer and tight junction proteins are two key factors to maintain regular intestinal epithelial barrier function. The mucus layer provides a physiochemical barrier to protect the epithelial cell surface. Previous studies indicated that anthocyanins-rich food consumption significantly increased the secretion of membrane-associated mucins and wound-enclosure proteins including MUC1, MUC2, MUC3, Cdc42, Rac1, GAL2, GAL3, GAL4, and RELMβ, which play a vital role in the mucus injury repair process [121,122]. Tight junctions establish the paracellular barrier that controls the flow of molecules in the intercellular space between epithelial cells. As the building blocks of epithelial tight junction, different TJ proteins play different roles. Claudin 1 and Claudin 4 contribute to the tightening of the epithelium, whereas Claudin 2 may be partially responsible for the luminal uptake of antigenic macromolecules because of induction of TJ strand discontinuities [123,124,125]. Occludin involved in cellular adhesion regulates paracellular permeability [126]. ZO-1, which is a classic TJ marker, functions as an “anchor” and is responsible for linking occludin, claudin, and actin cytoskeleton to enhance the epithelial barrier [127,128]. Anthocyanins from a purple-fleshed potato reduced the cell permeability *in vitro* using a Caco-2 cells [129]. In another study, mice were supplemented with 100 mg/kg black rice extract via oral gavage, and then provided with 2% DSS in their drinking water for five days to induce colitis. Mice on black rice supplementation showed a reduced histological score, which suggests alleviated mucosal injury and edema compared to DSS treatment [90]. In a DSS-induced murine colitis model, the cooked black bean diet (20%) consumption for two weeks significantly inhibited the colon shortening and spleen enlargement in mice [130]. Shima Bibi et al. evaluated the intestinal barrier protective activity of anthocyanins from red raspberries and reported that the red raspberries supplementation observably suppressed the elevation of claudin-2 protein and enhanced the expression of claudin-3 and ZO-1 under DSS treatment [122]. These above results indicate that anthocyanins can protect the tight junctions by modulating the ratio of TJ-positive and negative proteins and confirm the protective effect of anthocyanins from different fruits and vegetables against colonic inflammation [131].

### 3.2. Anthocyanins and Immunomodulation

**Figure 3:**
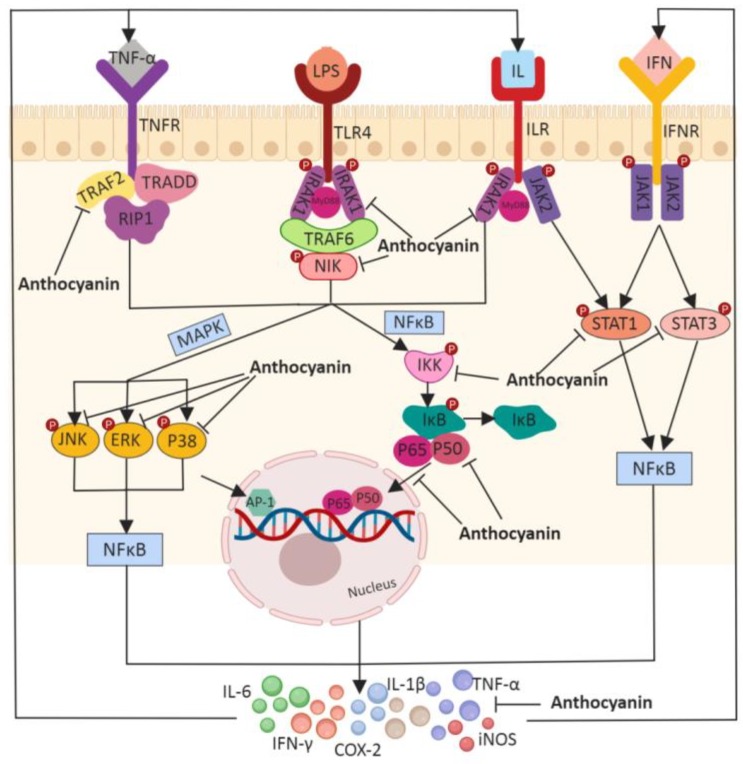
The mechanisms through which anthocyanins act as anti-inflammatory agents. Inflammatory signaling pathways including NF-kB, MAPKs (P38, ERK, JNK), and STATs were activated by ligand binding of the pro-inflammatory cytokines TNF-α, LPS, IL, and IFN, which eventually leads to the translocation of transcription factors to the nucleus, transcriptional activation, and cytokine production. Anthocyanins attenuated the cascade of inflammatory responses by inhibiting the translocation of transcription factors (P50 and P65), the phosphorylation of IRAK1, NIK, IKK, STAT1, STAT3, P38, ERK, and JNK, the secretion of inflammatory cytokines (IL-6, IL-1β, TNF-α, iNOS, COX-2, and IFN-γ), and activation of NF-kB, MAPK, and STAT inflammatory signaling pathways.

Anthocyanin-rich bilberry extract (ARBE) and single anthocyanin cyanidin-3-O-glycoside (C3G) application significantly inhibited the expression and secretion of TNF-α in stimulated human colon epithelial T84 cells [132]. Blueberry supplementation in an obesity-associated chronic inflammation rat model showed elevated production of acetate and reduced expression levels of TNF-α and IL-1β compared to control rats [133]. The protective effect of blueberry anthocyanin extract has also been confirmed in trinitrobenzene sulfonic acid (TNBS)-induced colitis mice model, where researchers found that anthocyanin treatment restored not only IL-10 secretion but also reduced serum levels of IL-12, TNF-α, and IFN-γ. In the same study, anthocyanin supplementation showed amelioration of morphological and histological symptoms of colitis in a dose-dependent manner [134]. In a recent study by Lei Zhao et al., mice supplemented with 100 mg/kg black rice extract via oral gavage showed a reduction in DSS-induced colonic IL-6, IL-1β, and TNF-α expression levels and MPO levels that are linearly related to the neutrophil infiltration [90]. Anthocyanin fraction from the tubers of purple yam down-regulated TNF-α, IFN-γ, and inflammation-associated ROS-producing enzyme myeloperoxidase (MPO) in mice treated with TNBS to induce colitis [135]. Similar observations are reported in a study using grapes, where anthocyanin-rich grape pomace extracts were found to prevent a DSS-induced increase of IL-6, MPO, and nitric oxide synthase (iNOS), whose production is triggered by bacterial products and pro-inflammatory cytokines [136]. Administration of purple-fleshed potatoes rich in malvidin and petunidin have shown to reduce the secretion of pro-inflammatory cytokines and, thereby, attenuate dextran sodium sulfate (DSS)-induced colitis in mice [88]. Anthocyanins also play a role in inhibiting chemokine release and the subsequent NF-κB signaling pathway (Figure 3). Cyanidin and C3G displayed a clear inhibitory effect on macrophage migration and pro-inflammatory chemokines monocyte chemoattractant protein-1 (MCP-1) and macrophage inflammatory protein-related protein-2 (MRP-2) *in vitro* [137]. The p-Coumaroyl anthocyanin mixture (contains petanin, peonanin, malvanin, and pelanin) extracted from a dark purple-fleshed potato cultivar Jayoung displayed an inhibitory effect on the transcriptional activity and translocation of NF-κB in RAW264.7 macrophages [138]. Another *in vitro* study reported that a pure sour cherry anthocyanin extract addition to human Caco-2 cells receded the translocation of a p65 subunit from the cytosol to nuclei [139]. Studies also linked the anti-inflammatory activity of anthocyanins to the inhibition of the COX-2 cascade. Both *in vivo* and *in vitro* evidence show that anthocyanins can suppress the expression level of COX-2 as well as the transactivation of AP-1, which is a transcription factor that regulates COX-2 gene expression [140,141]. Moreover, C3G can reduce COX-2 producing prostaglandin E2 (PGE2) production in human intestine HT-29 cells [142]. Additionally, a six-week ARBE treatment on UC patients revealed decreased serum levels of TNF-α, IFN-γ, and activated NF-κB subunit p65 and increased serum levels of IL-10 and IL-22 [143]. These results suggest that anthocyanins act as anti-inflammatory agents by their transcriptional and translational regulation of cytokines to inhibit/suppress pro-inflammatory cytokines and elevate the anti-inflammatory cytokines.

### 3.3. Anthocyanins and Gut Microbiota

The health-promoting effects of individual anthocyanins and their mixtures have been attributed not only to their direct effects in the colon but also to their metabolism by intestinal microbiota and their alteration of intestinal microbial populations. Anthocyanins and gut microbiota exhibit a two-way interaction to impact host physiology. Intestinal microbiota as a “metabolizing organ” plays a critical role in maintaining gastrointestinal health [144] and host metabolism [145,146]. Gut microbiota is a crucial determinant of anthocyanin bioavailability. 

In the lumen of the large intestine, unabsorbed anthocyanins are exposed to microbiota-mediated biotransformation, which includes three significant conditions: hydrolysis (breaking glycosidic linkages), fission (cleaving heterocycle), and demethylation. Bacterial species that carry corresponding β-glucosidase, β-glucuronidase, α-rhamnosidase, or demethylase such as *Clostridium* spp., *Butyrivibrio* spp., *Lactobacillus* spp., *B. fragilis*, and *B. ovatus*, etc., are actively involved in this process [147,148]. Anthocyanin biotransformation also produces glucose, which is an essential energy source required for bacterial growth [144]. Primary anthocyanin-derived metabolites are phenolic acids, whose anti-inflammatory effects have been verified by substantial studies. For example, the predominant metabolite of cyanidin and protocatechuic acid (PCA) has been shown to suppress COX-2 and iNOS protein expression and attenuate DSS-induced UC in mice [149]. Gallic acid as another anthocyanin-derived metabolite was shown to reduce the growth of potentially harmful bacteria such as *Clostridium histolyticum* and *Bacteroides* spp. without any negative effect on measured beneficial bacteria [150]. 

There is broad agreement that dietary anthocyanins and their metabolites have potential health benefits via modulation of the gut microbiota [44,150]. Increasing evidence supports the idea that anthocyanins can function as prebiotics, which contributes to the growth of certain commensal bacteria [44,151,152]. Both *in vitro* and *in vivo* studies have shown an elevated growth of potentially beneficial bacteria such as *Lactobacillus* spp. and *Bifidobacterium* spp. after administration of anthocyanin-rich products [44,151,152]. Anthocyanins can also interact with starch, SCFAs, and ferric iron to indirectly modulate gut microbiota. Anthocyanins exert the beneficial effect by increasing the levels of SCFAs, which has the antimicrobial impact on pathogens [153]. Moreover, it was found that anthocyanins were able to affect the digestion of starch by inhibiting digestive enzymes, such as α-amylase [154,155]. The indigestible starch goes down to the large intestine, where it can act as an energy source for several probiotic bacteria such as *lactobacilli*, *bifidobacteria*, and *streptococci*, which are beneficial to human health [155,156]. Another impressive result showed that indigestible dietary fiber components, such as β-glucans and resistant starch, can significantly increase the production of SCFAs [157,158]. Evidence indicated that the dysbiosis of the gut microbiota and impaired intestinal barrier function could be induced by Fe deficiency [159]. However, this situation can be alleviated with anthocyanin supplementation. Studies reported that C3G, cyanidin-3-5-diglucoside, petunidin-3-glucoside, and delphinidin-3-glucoside exerted substantial ferric ion chelating activities. Ferric ion chelation increases its solubility and bioavailability and may contribute to the intestinal homeostasis [160,161,162].

The above evidence demonstrated the anti-inflammatory properties of anthocyanins and the potential of anthocyanin to be used as novel therapeutic agents in UC treatment. Even though the mechanism behind anthocyanin-induced UC mitigation is not entirely known, it is highly likely that anthocyanin and bacteria interplay while anthocyanin-derived metabolites play a crucial role. There is no proven consensus regarding the bioavailability of anthocyanins, and minimal research has been done to elucidate the bioactivity of anthocyanins *in vivo*. Majority of studies focusing on the anti-colitis effect of anthocyanins utilize fruit or grain extract containing other bioactive compounds that are known to have an anti-oxidant effect. Thus, it is challenging to ascribe the observed UC relief to anthocyanins solely. Moreover, the possible synergistic effect of anthocyanins with other phytochemicals and fiber is a topic that requires more attention and effort to address the need for searching for a natural and safe anti-colitis strategy.

## Figures and Tables

**Table 1:** Bacterial metabolites of major anthocyanidins.

Chemical Class	Bacteria	Major Metabolites	Reference
Cyanidin	*Lachnospiraceae*, *Bifidobacteria*, and *Lactobacillus*.	Vanillic acid and protocatechuic acid	[41,42,43]
Peonidin		Vanillic acid and protocatechuic acid	[41,42]
Pelargonidin		4-hydroxybenzoic acid, hydroxycinnamic acid, p-coumaric acid, ferulic acid, and caffeic acid	[41,42]
Malvidin		Syringic acid, gallic acid, and pyrogallol	[44]
Delphinidin		Gallic acid and syringic acid	[41,42,45]
Petunidin		Gallic acid	[42]

**Table 2:** Sources of anthocyanins and their health benefits.

Chemical Class	Plant Source	Health Benefit	Reference
Cyanidin	Blueberries, bilberries, cranberries, elderberries, raspberry seeds, strawberries, purple corn, tea, purple carrot, purple rice	Anti-inflammatory and anti-cancer activity, prevention of cardiac disease, amelioration of perturbations in mitochondrial energy metabolism, and scavenging of reactive oxygen species as well as the promotion of neuronal plasticity.	[55,56,57,58,59]
Peonidin	Cranberry, blackcurrant, blueberry, huckleberry, bilberry, myrtles, roselle plants, purple-fleshed sweet potatoes, raw black rice, and centella asiatica	Antioxidative, anti-inflammatory, antimicrobial, antidiabetic, and cardioprotective effect.	[55,56,59,60]
Pelargonidin	Cranberry, verbena, strawberry, red corn, red potato	Cardiovascular disease prevention, obesity control, alleviation of diabetes, improvement of vision and memory, and increased immune defenses.	[61,62,63,64,65]
Malvidin	Red grape, blue pimpernel, cranberry, blueberries, saskatoon berries	Antioxidative, anti-inflammatory, and anti-cancer activity.	[66]
Delphinidin	Cranberry, Bilberry, Pomegranate, red potato, purple potato	Anti-inflammatory, prevention of bone loss, and anti-cancer activity.	[61,64,67,68,69,70]
Petunidin	Cranberry, grapes, black goji, color-fleshed potato, mango, bluberry, red banana, black bean	Antioxidative, anti-inflammatory, anti-diabetic, and neuroprotective effect.	[55,56,71,72,73,74,75,76,77]

## Abbreviations

APCs	Antigen-presenting cells
ARBE	Anthocyanin-rich bilberry extract
C3G	Cyanidin-3-glucoside
Cdc	Cell division control protein
COX-2	Cyclooxygenase-2
DCs	Dendritic cells
DSS	Dextran sodium sulfate
ERK	Extracellular signal-regulated kinase
GAL	Galectin
IBD	Inflammatory bowel disease
IFN-γ	Interferon gamma
IL	Interleukin
iNOS	Nitric oxide synthase
JNK	c-Jun N-terminal kinase
LPS	Lipopolysaccharide
MAPK	Mitogen-activated protein kinase
MCP-1	Chemoattractant protein-1
MPO	Myeloperoxidase
MRP-2	Macrophage inflammatory protein-related protein-2
MUC	Mucin
NF-κB	Nuclear factor-κB
PCA	Protocatechuic acid
PGE2	Prostaglandin E2
PSA	Polysaccharide A
RELMβ	Resistin-Like Molecule-beta
ROS	Reactive oxygen species
SCFA	Short chain fatty acid
STAT	Signal transducer and activator of transcription
Th	T-helper
TJ	Tight junction
TLR4	Toll-like receptors 4
TNBS	Trinitrobenzene sulfonic acid
TNFR	Tumor necrosis factor receptor
TNF-α	Tumor necrosis factor alpha
TRAF	TNF receptor-associated factor
Tregs	Regulatory T cells
UC	Ulcerative colitis
ZO-1	Zonula occludens-1

## References

[1] Wallace T.C., Giusti M.M. (2015). Anthocyanins. Adv. Nutr..

[2] Andersen O.M., Markham K.R. (2005). Flavonoids: Chemistry, biochemistry and applications.

[3] McGhie T.K., Walton M.C. (2007). The bioavailability and absorption of anthocyanins: Towards a better understanding. Mol. Nutr. Food Res..

[4] Borges G.D.S.C., Vieira F.G.K., Copetti C., Gonzaga L.V., Zambiazi R.C., Mancini Filho J., Fett R. (2011). Chemical characterization, bioactive compounds, and antioxidant capacity of jussara (euterpe edulis) fruit from the atlantic forest in southern brazil. Food Res. Int..

[5] Sui X., Zhang Y., Zhou W. (2016). Bread fortified with anthocyanin-rich extract from black rice as nutraceutical sources: Its quality attributes and in vitro digestibility. Food Chem..

[6] Morais C.A., de Rosso V.V., Estadella D., Pisani L.P. (2016). Anthocyanins as inflammatory modulators and the role of the gut microbiota. J. Nutr. Biochem..

[7] Pojer E., Mattivi F., Johnson D., Stockley C.S. (2013). The case for anthocyanin consumption to promote human health: A review. Compr. Rev. Food Sci. Food Saf..

[8] Welch C.R., Wu Q., Simon J.E. (2008). Recent advances in anthocyanin analysis and characterization. Curr. Anal. Chem..

[9] Pérez-Gregorio R.M., García-Falcón M.S., Simal-Gándara J., Rodrigues A.S., Almeida D.P. (2010). Identification and quantification of flavonoids in traditional cultivars of red and white onions at harvest. J. Food Compos. Anal..

[10] Woodward G., Kroon P., Cassidy A., Kay C. (2009). Anthocyanin stability and recovery: Implications for the analysis of clinical and experimental samples. J. Agric. Food Chem..

[11] Khoo H.E., Azlan A., Tang S.T., Lim S.M. (2017). Anthocyanidins and anthocyanins: Colored pigments as food, pharmaceutical ingredients, and the potential health benefits. Food Nutr. Res..

[12] Brouillard R. (1982). Chemical structure of anthocyanins.

[13] He J., Giusti M.M. (2010). Anthocyanins: Natural colorants with health-promoting properties. Annu. Rev. Food Sci. Technol..

[14] Wrolstad R.E., Durst R.W., Lee J. (2005). Tracking color and pigment changes in anthocyanin products. Trends Food Sci. Technol..

[15] Samadi A.K., Bilsland A., Georgakilas A.G., Amedei A., Amin A., Bishayee A., Azmi A.S., Lokeshwar B.L., Grue B., Panis C. (2015). Seminars in cancer biology. A Multi-Targeted Approach to Suppress Tumor-Promoting Inflammation.

[16] Andersen Ø.M., Jordheim M. (2013). Basic anthocyanin chemistry and dietary sources. Anthocyanins Health Dis..

[17] Smeriglio A., Barreca D., Bellocco E., Trombetta D. (2016). Chemistry, pharmacology and health benefits of anthocyanins. Phytother. Res..

[18] de Pascual-Teresa S., Sanchez-Ballesta M.T. (2008). Anthocyanins: From plant to health. Phytochem. Rev..

[19] Fang J. (2014). Bioavailability of anthocyanins. Drug Metab. Rev..

[20] Kong J.-M., Chia L.-S., Goh N.-K., Chia T.-F., Brouillard R. (2003). Analysis and biological activities of anthocyanins. Phytochemistry.

[21] Nayak C.A., Srinivas P., Rastogi N.K. (2010). Characterisation of anthocyanins from garcinia indica choisy. Food Chem..

[22] da Silva F.L., Escribano-Bailón M.T., Alonso J.J.P., Rivas-Gonzalo J.C., Santos-Buelga C. (2007). Anthocyanin pigments in strawberry. Lwt-Food Sci. Technol..

[23] Böhm H.G. (1994). Mazza und E. Miniati: Anthocyanins in Fruits, Vegetables and Grains. 362 Seiten, zahlr. Abb. und Tab. CRC Press: Boca Raton, Ann Arbor, London, Tokyo 1993. Preis: 144—£. Food Nahrung.

[24] Zamora-Ros R., Knaze V., Luján-Barroso L., Slimani N., Romieu I., Touillaud M., Kaaks R., Teucher B., Mattiello A., Grioni S. (2011). Estimation of the intake of anthocyanidins and their food sources in the european prospective investigation into cancer and nutrition (epic) study. Br. J. Nutr..

[25] Sebastian R.S., Wilkinson Enns C., Goldman J.D., Martin C.L., Steinfeldt L.C., Murayi T., Moshfegh A.J. (2015). A new database facilitates characterization of flavonoid intake, sources, and positive associations with diet quality among us adults. J. Nutr..

[26] Yousuf B., Gul K., Wani A.A., Singh P. (2016). Health benefits of anthocyanins and their encapsulation for potential use in food systems: A review. Crit. Rev. Food Sci. Nutr..

[27] Yi W., Akoh C.C., Fischer J., Krewer G. (2006). Absorption of anthocyanins from blueberry extracts by caco-2 human intestinal cell monolayers. J. Agric. Food Chem..

[28] Tsuda T., Shiga K., Ohshima K., Kawakishi S., Osawa T. (1996). Inhibition of lipid peroxidation and the active oxygen radical scavenging effect of anthocyanin pigments isolated from phaseolus vulgaris l. Biochem. Pharmacol..

[29] Zhang Y., Vareed S.K., Nair M.G. (2005). Human tumor cell growth inhibition by nontoxic anthocyanidins, the pigments in fruits and vegetables. Life Sci..

[30] Milbury P.E., Cao G., Prior R.L., Blumberg J. (2002). Bioavailablility of elderberry anthocyanins. Mech. Ageing Dev..

[31] Talavera S., Felgines C., Texier O., Besson C., Lamaison J.-L., Rémésy C. (2003). Anthocyanins are efficiently absorbed from the stomach in anesthetized rats. J. Nutr..

[32] Matuschek M.C., Hendriks W.H., McGhie T.K., Reynolds G.W. (2006). The jejunum is the main site of absorption for anthocyanins in mice. J. Nutr. Biochem..

[33] Czank C., Cassidy A., Zhang Q., Morrison D.J., Preston T., Kroon P.A., Botting N.P., Kay C.D. (2013). Human metabolism and elimination of the anthocyanin, cyanidin-3-glucoside: A 13c-tracer study. Am. Clin. Nutr..

[34] Felgines C., Krisa S., Mauray A., Besson C., Lamaison J.-L., Scalbert A., Mérillon J.-M., Texier O. (2010). Radiolabelled cyanidin 3-o-glucoside is poorly absorbed in the mouse. Br. J. Nutr..

[35] Bub A., Watzl B., Heeb D., Rechkemmer G., Briviba K. (2001). Malvidin-3-glucoside bioavailability in humans after ingestion of red wine, dealcoholized red wine and red grape juice. Eur. J. Nutr..

[36] Matsumoto H., Inaba H., Kishi M., Tominaga S., Hirayama M., Tsuda T. (2001). Orally administered delphinidin 3-rutinoside and cyanidin 3-rutinoside are directly absorbed in rats and humans and appear in the blood as the intact forms. J. Agric. Food Chem..

[37] Manach C., Williamson G., Morand C., Scalbert A., Rémésy C. (2005). Bioavailability and bioefficacy of polyphenols in humans. I. Review of 97 bioavailability studies. Am. J. Clin. Nutr..

[38] Aura A.-M., Martin-Lopez P., O’Leary K.A., Williamson G., Oksman-Caldentey K.-M., Poutanen K., Santos-Buelga C. (2005). In vitro metabolism of anthocyanins by human gut microflora. Eur. J. Nutr..

[39] Keppler K., Humpf H.-U. (2005). Metabolism of anthocyanins and their phenolic degradation products by the intestinal microflora. Bioorganic Med. Chem..

[40] Vamanu E., Gatea F., Sârbu I., Pelinescu D. (2019). An in vitro study of the influence of curcuma longa extracts on the microbiota modulation process, in patients with hypertension. Pharmaceutics.

[41] Fleschhut J., Kratzer F., Rechkemmer G., Kulling S.E. (2006). Stability and biotransformation of various dietary anthocyanins in vitro. Eur. J. Nutr..

[42] Forester S.C., Waterhouse A.L. (2008). Identification of cabernet sauvignon anthocyanin gut microflora metabolites. J. Agric. Food Chem..

[43] Salyer J., Park S., Ricke S., Lee S. (2013). Analysis of microbial populations and metabolism of anthocyanins by mice gut microflora fed with blackberry powder. J. Nutr. Food Sci..

[44] Hidalgo M., Oruna-Concha M.J., Kolida S., Walton G.E., Kallithraka S., Spencer J.P., de Pascual-Teresa S. (2012). Metabolism of anthocyanins by human gut microflora and their influence on gut bacterial growth. J. Agric. Food Chem..

[45] Chen Y., Li Q., Zhao T., Zhang Z., Mao G., Feng W., Wu X., Yang L. (2017). Biotransformation and metabolism of three mulberry anthocyanin monomers by rat gut microflora. Food Chem..

[46] Zielińska M., Lewandowska U., Podsędek A., Cygankiewicz A.I., Jacenik D., Sałaga M., Kordek R., Krajewska W.M., Fichna J. (2015). Orally available extract from brassica oleracea var. Capitata rubra attenuates experimental colitis in mouse models of inflammatory bowel diseases. J. Funct. Foods.

[47] Sugata M., Lin C.-Y., Shih Y.-C. (2015). Anti-inflammatory and anticancer activities of taiwanese purple-fleshed sweet potatoes (ipomoea batatas l. Lam) extracts. Biomed. Res. Int..

[48] Medda R., Lyros O., Schmidt J.L., Jovanovic N., Nie L., Link B.J., Otterson M.F., Stoner G.D., Shaker R., Rafiee P. (2015). Anti inflammatory and anti angiogenic effect of black raspberry extract on human esophageal and intestinal microvascular endothelial cells. Microvasc. Res..

[49] Youdim K.A., Shukitt-Hale B., Joseph J.A. (2004). Flavonoids and the brain: Interactions at the blood–brain barrier and their physiological effects on the central nervous system. Free Radic. Biol. Med..

[50] Cassidy A., Mukamal K.J., Liu L., Franz M., Eliassen A.H., Rimm E.B. (2013). High anthocyanin intake is associated with a reduced risk of myocardial infarction in young and middle-aged women. Circulation.

[51] Mink P.J., Scrafford C.G., Barraj L.M., Harnack L., Hong C.-P., Nettleton J.A., Jacobs D.R. (2007). Flavonoid intake and cardiovascular disease mortality: A prospective study in postmenopausal women. Am. J. Clin. Nutr..

[52] Vendrame S., Del Bo C., Ciappellano S., Riso P., Klimis-Zacas D. (2016). Berry fruit consumption and metabolic syndrome. Antioxidants.

[53] Overall J., Bonney S., Wilson M., Beermann A., Grace M., Esposito D., Lila M., Komarnytsky S. (2017). Metabolic effects of berries with structurally diverse anthocyanins. Int. J. Mol. Sci..

[54] J Thoppil R., Bhatia D., F Barnes K., Haznagy-Radnai E., Hohmann J., S Darvesh A., Bishayee A. (2012). Black currant anthocyanins abrogate oxidative stress through nrf2-mediated antioxidant mechanisms in a rat model of hepatocellular carcinoma. Curr. Cancer Drug Targets.

[55] Wu X., Prior R.L. (2005). Systematic identification and characterization of anthocyanins by hplc-esi-ms/ms in common foods in the united states: Fruits and berries. J. Agric. Food Chem..

[56] Khoo C., Falk M. (2014). Cranberry polyphenols: Effects on cardiovascular risk factors. Polyphenols in human health and disease.

[57] Rothenberg D.O., Yang H., Chen M., Zhang W., Zhang L. (2019). Metabolome and transcriptome sequencing analysis reveals anthocyanin metabolism in pink flowers of anthocyanin-rich tea (camellia sinensis). Molecules.

[58] Tsutsumi A., Horikoshi Y., Fushimi T., Saito A., Koizumi R., Fujii Y., Hu Q.Q., Hirota Y., Aizawa K., Osakabe N. (2019). Acylated anthocyanins derived from purple carrot (daucus carota l.) induce elevation of blood flow in rat cremaster arteriole. Food Funct..

[59] Wongwichai T., Teeyakasem P., Pruksakorn D., Kongtawelert P., Pothacharoen P. (2019). Anthocyanins and metabolites from purple rice inhibit il-1beta-induced matrix metalloproteinases expression in human articular chondrocytes through the nf-kappab and erk/mapk pathway. Biomed. Pharmacother..

[60] Jayaprakasam B., Vareed S.K., Olson L.K., Nair M.G. (2005). Insulin secretion by bioactive anthocyanins and anthocyanidins present in fruits. J. Agric. Food Chem..

[61] Amini A.M., Muzs K., Spencer J.P., Yaqoob P. (2017). Pelargonidin-3-o-glucoside and its metabolites have modest anti-inflammatory effects in human whole blood cultures. Nutr. Res..

[62] Tsuda T. (2012). Dietary anthocyanin-rich plants: Biochemical basis and recent progress in health benefits studies. Mol. Nutr. Food Res..

[63] Rodriguez-Mateos A., Heiss C., Borges G., Crozier A. (2013). Berry (poly) phenols and cardiovascular health. J. Agric. Food Chem..

[64] Stushnoff C., Holm D., Thompson M.D., Jiang W., Thompson H.J., Joyce N.I., Wilson P. (2008). Antioxidant properties of cultivars and selections from the colorado potato breeding program. Am. J. Potato Res..

[65] Nankar A.N., Dungan B., Paz N., Sudasinghe N., Schaub T., Holguin F.O., Pratt R.C. (2016). Quantitative and qualitative evaluation of kernel anthocyanins from southwestern united states blue corn. J. Sci Food Agric..

[66] Bognar E., Sarszegi Z., Szabo A., Debreceni B., Kalman N., Tucsek Z., Sumegi B., Gallyas F. (2013). Antioxidant and anti-inflammatory effects in raw264. 7 macrophages of malvidin, a major red wine polyphenol. PLoS ONE.

[67] Moriwaki S., Suzuki K., Muramatsu M., Nomura A., Inoue F., Into T., Yoshiko Y., Niida S. (2014). Delphinidin, one of the major anthocyanidins, prevents bone loss through the inhibition of excessive osteoclastogenesis in osteoporosis model mice. PLoS ONE.

[68] Hafeez B.B., Siddiqui I.A., Asim M., Malik A., Afaq F., Adhami V.M., Saleem M., Din M., Mukhtar H. (2008). A dietary anthocyanidin delphinidin induces apoptosis of human prostate cancer pc3 cells in vitro and in vivo: Involvement of nuclear factor-κb signaling. Cancer Res..

[69] Spilmont M., Léotoing L., Davicco M.J., Lebecque P., Miot-Noirault E., Pilet P., Rios L., Wittrant Y., Coxam V. (2015). Pomegranate peel extract prevents bone loss in a preclinical model of osteoporosis and stimulates osteoblastic differentiation in vitro. Nutrients.

[70] Lao F., Sigurdson G.T., Giusti M.M. (2017). Health benefits of purple corn (zea mays l.) phenolic compounds. Compr. Rev. Food Sci. Food Saf..

[71] Muche B.M., Speers R.A., Rupasinghe H.P.V. (2018). Storage temperature impacts on anthocyanins degradation, color changes and haze development in juice of "merlot" and "ruby" grapes (vitis vinifera). Front. Nutr..

[72] Tang P., Giusti M.M. (2018). Black goji as a potential source of natural color in a wide ph range. Food Chem..

[73] Rocha-Parra D., Chirife J., Zamora C., de Pascual-Teresa S. (2018). Chemical characterization of an encapsulated red wine powder and its effects on neuronal cells. Molecules.

[74] Kalita D., Holm D.G., LaBarbera D.V., Petrash J.M., Jayanty S.S. (2018). Inhibition of alpha-glucosidase, alpha-amylase, and aldose reductase by potato polyphenolic compounds. PLoS ONE.

[75] Lopez-Cobo A., Verardo V., Diaz-de-Cerio E., Segura-Carretero A., Fernandez-Gutierrez A., Gomez-Caravaca A.M. (2017). Use of hplc- and gc-qtof to determine hydrophilic and lipophilic phenols in mango fruit (mangifera indica l.) and its by-products. Food Res. Int..

[76] Fu X., Cheng S., Liao Y., Huang B., Du B., Zeng W., Jiang Y., Duan X., Yang Z. (2018). Comparative analysis of pigments in red and yellow banana fruit. Food Chem..

[77] Aguilera Y., Mojica L., Rebollo-Hernanz M., Berhow M., de Mejia E.G., Martin-Cabrejas M.A. (2016). Black bean coats: New source of anthocyanins stabilized by beta-cyclodextrin copigmentation in a sport beverage. Food Chem..

[78] Adams S.M., Bornemann P.H. (2013). Ulcerative colitis. Am. Fam. Physician.

[79] Burisch J., Pedersen N., Čuković-Čavka S., Brinar M., Kaimakliotis I., Duricova D., Shonová O., Vind I., Avnstrøm S., Thorsgaard N. (2014). East–west gradient in the incidence of inflammatory bowel disease in europe: The ecco-epicom inception cohort. Gut.

[80] Cosnes J., Gower–Rousseau C., Seksik P., Cortot A. (2011). Epidemiology and natural history of inflammatory bowel diseases. Gastroenterology.

[81] Jess T., Simonsen J., Nielsen N.M., Jørgensen K.T., Bager P., Ethelberg S., Frisch M. (2011). Enteric salmonella or campylobacter infections and the risk of inflammatory bowel disease. Gut.

[82] Sahami S., Kooij I., Meijer S., Van den Brink G., Buskens C., Te Velde A. (2016). The link between the appendix and ulcerative colitis: Clinical relevance and potential immunological mechanisms. Am. J. Gastroenterol..

[83] Hou J.K., Abraham B., El-Serag H. (2011). Dietary intake and risk of developing inflammatory bowel disease: A systematic review of the literature. Am. J. Gastroenterol..

[84] Ananthakrishnan A.N., Higuchi L.M., Huang E.S., Khalili H., Richter J.M., Fuchs C.S., Chan A.T. (2012). Aspirin, nonsteroidal anti-inflammatory drug use, and risk for crohn disease and ulcerative colitis: A cohort study. Ann. Intern. Med..

[85] Khalili H., Higuchi L.M., Ananthakrishnan A.N., Manson J.E., Feskanich D., Richter J.M., Fuchs C.S., Chan A.T. (2012). Hormone therapy increases risk of ulcerative colitis but not crohn’s disease. Gastroenterology.

[86] Ungaro R., Bernstein C.N., Gearry R., Hviid A., Kolho K.-L., Kronman M.P., Shaw S., Van Kruiningen H., Colombel J.-F., Atreja A. (2014). Antibiotics associated with increased risk of new-onset crohn’s disease but not ulcerative colitis: A meta-analysis. Am. J. Gastroenterol..

[87] Chen L., Zhou Z., Yang Y., Chen N., Xiang H. (2017). Therapeutic effect of imiquimod on dextran sulfate sodium-induced ulcerative colitis in mice. PLoS ONE.

[88] Reddivari L., Wang T., Wu B., Li S. (2019). Potato: An Anti-Inflammatory Food. Am. J. Potato Res..

[89] Johansson M.E., Gustafsson J.K., Sjöberg K.E., Petersson J., Holm L., Sjövall H., Hansson G.C. (2010). Bacteria penetrate the inner mucus layer before inflammation in the dextran sulfate colitis model. PLoS ONE.

[90] Zhao L., Zhang Y., Liu G., Hao S., Wang C., Wang Y. (2018). Black rice anthocyanin-rich extract and rosmarinic acid, alone and in combination, protect against dss-induced colitis in mice. Food Funct..

[91] Minaiyan M., Ghannadi A., Mahzouni P., Jaffari-Shirazi E. (2011). Comparative study of berberis vulgaris fruit extract and berberine chloride effects on acetic acid-induced colitis in rats. Iran. J. Pharm. Res. Ijpr.

[92] Atreya R., Mudter J., Finotto S., Müllberg J., Jostock T., Wirtz S., Schütz M., Bartsch B., Holtmann M., Becker C. (2000). Blockade of interleukin 6 trans signaling suppresses t-cell resistance against apoptosis in chronic intestinal inflammation: Evidence in crohn disease and experimental colitis in vivo. Nat. Med..

[93] Neurath M.F. (2014). Cytokines in inflammatory bowel disease. Nat. Rev. Immunol..

[94] Su L., Nalle S.C., Shen L., Turner E.S., Singh G., Breskin L.A., Khramtsova E.A., Khramtsova G., Tsai P.Y., Fu Y.X. (2013). Tnfr2 activates mlck-dependent tight junction dysregulation to cause apoptosis-mediated barrier loss and experimental colitis. Gastroenterology.

[95] Hernández-Chirlaque C., Aranda C.J., Ocón B., Capitán-Cañadas F., Ortega-González M., Carrero J.J., Suárez M.D., Zarzuelo A., Sánchez de Medina F., Martínez-Augustin O. (2016). Germ-free and antibiotic-treated mice are highly susceptible to epithelial injury in dss colitis. J. Crohn’s Colitis.

[96] Bernardo D., Vallejo-Díez S., Mann E.R., Al-Hassi H.O., Martínez-Abad B., Montalvillo E., Tee C.T., Murugananthan A.U., Núñez H., Peake S.T. (2012). Il-6 promotes immune responses in human ulcerative colitis and induces a skin-homing phenotype in the dendritic cells and t cells they stimulate. Eur. J. Immunol..

[97] Ono Y., Kanai T., Sujino T., Nemoto Y., Kanai Y., Mikami Y., Hayashi A., Matsumoto A., Takaishi H., Ogata H. (2012). T-helper 17 and interleukin-17–producing lymphoid tissue inducer-like cells make different contributions to colitis in mice. Gastroenterology.

[98] Wu Y.-D., Zhou B. (2010). Tnf-α/nf-κb/snail pathway in cancer cell migration and invasion. Br. J. Cancer.

[99] Hunter C.A., Jones S.A. (2015). Il-6 as a keystone cytokine in health and disease. Nat. Immunol..

[100] Stumhofer J.S., Silver J.S., Laurence A., Porrett P.M., Harris T.H., Turka L.A., Ernst M., Saris C.J., O’Shea J.J., Hunter C.A. (2007). Interleukins 27 and 6 induce stat3-mediated t cell production of interleukin 10. Nat. Immunol..

[101] Heller F., Florian P., Bojarski C., Richter J., Christ M., Hillenbrand B., Mankertz J., Gitter A.H., Bürgel N., Fromm M. (2005). Interleukin-13 is the key effector th2 cytokine in ulcerative colitis that affects epithelial tight junctions, apoptosis, and cell restitution. Gastroenterology.

[102] Pickert G., Neufert C., Leppkes M., Zheng Y., Wittkopf N., Warntjen M., Lehr H.-A., Hirth S., Weigmann B., Wirtz S. (2009). Stat3 links il-22 signaling in intestinal epithelial cells to mucosal wound healing. J. Exp. Med..

[103] Ordas I., Eckmann L., Talamini M., Baumgart D.C., Sandborn W.J. (2012). Ulcerative colitis. Lancet.

[104] Liu T., Zhang L., Joo D., Sun S.C. (2017). Nf-kappab signaling in inflammation. Signal. Transduct Target..

[105] Quigley E.M. (2013). Gut bacteria in health and disease. Gastroenterol. Hepatol..

[106] Mazmanian S.K., Round J.L., Kasper D.L. (2008). A microbial symbiosis factor prevents intestinal inflammatory disease. Nature.

[107] Lindemann R.K., Gabrielli B., Johnstone R.W. (2004). Histone-deacetylase inhibitors for the treatment of cancer. Cell Cycle.

[108] Smith P.M., Howitt M.R., Panikov N., Michaud M., Gallini C.A., Bohlooly-y M., Glickman J.N., Garrett W.S. (2013). The microbial metabolites, short-chain fatty acids, regulate colonic treg cell homeostasis. Science.

[109] Matsuoka K., Uemura Y., Kanai T., Kunisaki R., Suzuki Y., Yokoyama K., Yoshimura N., Hibi T. (2018). Efficacy of bifidobacterium breve fermented milk in maintaining remission of ulcerative colitis. Dig. Dis. Sci..

[110] Tamaki H., Nakase H., Inoue S., Kawanami C., Itani T., Ohana M., Kusaka T., Uose S., Hisatsune H., Tojo M. (2016). Efficacy of probiotic treatment with bifidobacterium longum 536 for induction of remission in active ulcerative colitis: A randomized, double-blinded, placebo-controlled multicenter trial. Dig. Endosc..

[111] Zocco M., Dal Verme L.Z., Cremonini F., Piscaglia A., Nista E., Candelli M., Novi M., Rigante D., Cazzato I., Ojetti V. (2006). Efficacy of lactobacillus gg in maintaining remission of ulcerative colitis. Aliment. Pharmacol. Ther..

[112] Shen Z.-H., Zhu C.-X., Quan Y.-S., Yang Z.-Y., Wu S., Luo W.-W., Tan B., Wang X.-Y. (2018). Relationship between intestinal microbiota and ulcerative colitis: Mechanisms and clinical application of probiotics and fecal microbiota transplantation. World J. Gastroenterol..

[113] Ott S., Musfeldt M., Wenderoth D., Hampe J., Brant O., Fölsch U., Timmis K., Schreiber S. (2004). Reduction in diversity of the colonic mucosa associated bacterial microflora in patients with active inflammatory bowel disease. Gut.

[114] Morgan X.C., Tickle T.L., Sokol H., Gevers D., Devaney K.L., Ward D.V., Reyes J.A., Shah S.A., LeLeiko N., Snapper S.B. (2012). Dysfunction of the intestinal microbiome in inflammatory bowel disease and treatment. Genome Biol..

[115] Sokol H., Lepage P., Seksik P., Dore J., Marteau P. (2006). Temperature gradient gel electrophoresis of fecal 16s rrna reveals active escherichia coli in the microbiota of patients with ulcerative colitis. J. Clin. Microbiol..

[116] Obiso R., Azghani A.O., Wilkins T.D. (1997). The bacteroides fragilis toxin fragilysin disrupts the paracellular barrier of epithelial cells. Infect. Immun..

[117] Wells C.L., Van de Westerlo E., Jechorek R.P., Feltis B., Wilkins T., Erlandsen S. (1996). Bacteroides fragilis enterotoxin modulates epithelial permeability and bacterial internalization by ht-29 enterocytes. Gastroenterology.

[118] Akiyama S., Nesumi A., Maeda-Yamamoto M., Uehara M., Murakami A. (2012). Effects of anthocyanin-rich tea “sunrouge” on dextran sodium sulfate-induced colitis in mice. BioFactors.

[119] Biedermann L., Mwinyi J., Scharl M., Frei P., Zeitz J., Kullak-Ublick G.A., Vavricka S.R., Fried M., Weber A., Humpf H.-U. (2013). Bilberry ingestion improves disease activity in mild to moderate ulcerative colitis—an open pilot study. J. Crohn’s Colitis.

[120] Kim J.-M., Kim J.-S., Yoo H., Choung M.-G., Sung M.-K. (2008). Effects of black soybean [glycine max (l.) merr.] seed coats and its anthocyanidins on colonic inflammation and cell proliferation in vitro and in vivo. J. Agric. Food Chem..

[121] Monk J.M., Wu W., Hutchinson A.L., Pauls P., Robinson L.E., Power K.A. (2018). Navy and black bean supplementation attenuates colitis-associated inflammation and colonic epithelial damage. J. Nutr. Biochem..

[122] Bibi S., Kang Y., Du M., Zhu M.-J. (2018). Dietary red raspberries attenuate dextran sulfate sodium-induced acute colitis. J. Nutr. Biochem..

[123] Turksen K., Troy T.-C. (2004). Barriers built on claudins. J. Cell Sci..

[124] Morita K., Furuse M., Fujimoto K., Tsukita S. (1999). Claudin multigene family encoding four-transmembrane domain protein components of tight junction strands. Proc. Natl. Acad. Sci. USA.

[125] Al-Asmakh M., Hedin L. (2015). Microbiota and the control of blood-tissue barriers. Tissue Barriers.

[126] Feldman G.J., Mullin J.M., Ryan M.P. (2005). Occludin: Structure, function and regulation. Adv. Drug Deliv. Rev..

[127] Umeda K., Matsui T., Nakayama M., Furuse K., Sasaki H., Furuse M., Tsukita S. (2004). Establishment and characterization of cultured epithelial cells lacking expression of zo-1. J. Biol. Chem..

[128] Groschwitz K.R., Hogan S.P. (2009). Intestinal barrier function: Molecular regulation and disease pathogenesis. J. Allergy Clin. Immunol..

[129] Sun X., Du M., Navarre D.A., Zhu M.J. (2018). Purple potato extract promotes intestinal epithelial differentiation and barrier function by activating amp-activated protein kinase. Mol. Nutr. Food Res..

[130] Zhang C., Monk J.M., Lu J.T., Zarepoor L., Wu W., Liu R., Pauls K.P., Wood G.A., Robinson L., Tsao R. (2014). Cooked navy and black bean diets improve biomarkers of colon health and reduce inflammation during colitis. Br. J. Nutr..

[131] Shan Q., Zheng Y., Lu J., Zhang Z., Wu D., Fan S., Hu B., Cai X., Cai H., Liu P. (2014). Purple sweet potato color ameliorates kidney damage via inhibiting oxidative stress mediated nlrp3 inflammasome activation in high fat diet mice. Food Chem. Toxicol..

[132] Triebel S., Trieu H.-L., Richling E. (2012). Modulation of inflammatory gene expression by a bilberry (vaccinium myrtillus l.) extract and single anthocyanins considering their limited stability under cell culture conditions. J. Agric. Food Chem..

[133] Fischer J.G., Keirsey K.I., Kirkland R., Lee S., Grunewald Z.I., de La Serre C.B. (2018). Blueberry supplementation influences the gut microbiota, inflammation, and insulin resistance in high-fat-diet–fed rats. J. Nutr..

[134] Wu L.H., Xu Z.L., Dong D., He S.A., Yu H. (2011). Protective effect of anthocyanins extract from blueberry on tnbs-induced ibd model of mice. Evid.-Based Complementary Altern. Med. Ecam.

[135] Chen T., Hu S., Zhang H., Guan Q., Yang Y., Wang X. (2017). Anti-inflammatory effects of dioscorea alata l. Anthocyanins in a tnbs-induced colitis model. Food Funct..

[136] Boussenna A., Cholet J., Goncalves-Mendes N., Joubert-Zakeyh J., Fraisse D., Vasson M.P., Texier O., Felgines C. (2016). Polyphenol-rich grape pomace extracts protect against dextran sulfate sodium-induced colitis in rats. J. Sci. Food Agric..

[137] Choe M.-R., Ji Hye K., Yoo H., Yang C.-H., Kim M.-O., Yu R.-N., Choe S.-Y. (2007). Cyanidin and cyanidin-3-o-β-d-glucoside suppress the inflammatory responses of obese adipose tissue by inhibiting the release of chemokines mcp-1 and mrp-2. J. Food Sci. Nutr..

[138] Lee H.H., Lee S.G., Shin J.S., Lee H.Y., Yoon K., Ji Y.W., Jang D.S., Lee K.T. (2017). P-coumaroyl anthocyanin mixture isolated from tuber epidermis of solanum tuberosum attenuates reactive oxygen species and pro-inflammatory mediators by suppressing nf-kappab and stat1/3 signaling in lps-induced raw264.7 macrophages. Biol. Pharm. Bull..

[139] Le Phuong Nguyen T., Fenyvesi F., Remenyik J., Homoki J.R., Gogolak P., Bacskay I., Feher P., Ujhelyi Z., Vasvari G., Vecsernyes M. (2018). Protective effect of pure sour cherry anthocyanin extract on cytokine-induced inflammatory caco-2 monolayers. Nutrients.

[140] Jung S.K., Lim T.-G., Seo S.G., Lee H.J., Hwang Y.-S., Choung M.-G., Lee K.W. (2013). Cyanidin-3-o-(2″-xylosyl)-glucoside, an anthocyanin from siberian ginseng (acanthopanax senticosus) fruits, inhibits uvb-induced cox-2 expression and ap-1 transactivation. Food Sci. Biotechnol..

[141] Li L., Wang L., Wu Z., Yao L., Wu Y., Huang L., Liu K., Zhou X., Gou D. (2014). Anthocyanin-rich fractions from red raspberries attenuate inflammation in both raw264.7 macrophages and a mouse model of colitis. Sci. Rep..

[142] Pereira S.R., Pereira R., Figueiredo I., Freitas V., Dinis T.C., Almeida L.M. (2017). Comparison of anti-inflammatory activities of an anthocyanin-rich fraction from portuguese blueberries (vaccinium corymbosum l.) and 5-aminosalicylic acid in a tnbs-induced colitis rat model. PLoS ONE.

[143] Roth S., Spalinger M.R., Gottier C., Biedermann L., Zeitz J., Lang S., Weber A., Rogler G., Scharl M. (2016). Bilberry-derived anthocyanins modulate cytokine expression in the intestine of patients with ulcerative colitis. PLoS ONE.

[144] Faria A., Fernandes I., Norberto S., Mateus N., Calhau C.A.O. (2014). Interplay between anthocyanins and gut microbiota. J. Agric. Food Chem..

[145] Ley R.E., Peterson D.A., Gordon J.I. (2006). Ecological and evolutionary forces shaping microbial diversity in the human intestine. Cell.

[146] Pan P., Lam V., Salzman N., Huang Y.-W., Yu J., Zhang J., Wang L.-S. (2017). Black raspberries and their anthocyanin and fiber fractions alter the composition and diversity of gut microbiota in f-344 rats. Nutr. Cancer.

[147] Cassidy A., Minihane A.M. (2017). The role of metabolism (and the microbiome) in defining the clinical efficacy of dietary flavonoids. Am. J. Clin. Nutr.

[148] Selma M.V., Espin J.C., Tomas-Barberan F.A. (2009). Interaction between phenolics and gut microbiota: Role in human health. J. Agric. Food Chem..

[149] Farombi E.O., Adedara I.A., Awoyemi O.V., Njoku C.R., Micah G.O., Esogwa C.U., Owumi S.E., Olopade J.O. (2016). Dietary protocatechuic acid ameliorates dextran sulphate sodium-induced ulcerative colitis and hepatotoxicity in rats. Food Funct..

[150] Parkar S.G., Trower T.M., Stevenson D.E. (2013). Fecal microbial metabolism of polyphenols and its effects on human gut microbiota. Anaerobe.

[151] Molan A.-L., Liu Z., Kruger M. (2010). The ability of blackcurrant extracts to positively modulate key markers of gastrointestinal function in rats. World J. Microbiol. Biotechnol..

[152] Bialonska D., Ramnani P., Kasimsetty S.G., Muntha K.R., Gibson G.R., Ferreira D. (2010). The influence of pomegranate by-product and punicalagins on selected groups of human intestinal microbiota. Int. J. Food Microbiol..

[153] Gibson G., Wang X. (1994). Regulatory effects of bifidobacteria on the growth of other colonic bacteria. J. Appl. Bacteriol..

[154] Miao M., Jiang H., Jiang B., Zhang T., Cui S.W., Jin Z. (2014). Phytonutrients for controlling starch digestion: Evaluation of grape skin extract. Food Chem..

[155] Camelo-Méndez G.A., Agama-Acevedo E., Sanchez-Rivera M.M., Bello-Pérez L.A. (2016). Effect on in vitro starch digestibility of mexican blue maize anthocyanins. Food Chem..

[156] Magallanes-Cruz P.A., Flores-Silva P.C., Bello-Perez L.A. (2017). Starch structure influences its digestibility: A review. J. Food Sci..

[157] Immerstrand T., Andersson K.E., Wange C., Rascon A., Hellstrand P., Nyman M., Cui S.W., Bergenståhl B., Trägårdh C., Öste R. (2010). Effects of oat bran, processed to different molecular weights of β-glucan, on plasma lipids and caecal formation of scfa in mice. Br. J. Nutr..

[158] Immerstrand T. (2010). Cholesterol-lowering properties of oats: Effects of processing and the role of oat components.

[159] Dostal A., Fehlbaum S., Chassard C., Zimmermann M.B., Lacroix C. (2013). Low iron availability in continuous in vitro colonic fermentations induces strong dysbiosis of the child gut microbial consortium and a decrease in main metabolites. Fems Microbiol. Ecol..

[160] Xie Y., Zhu X., Li Y., Wang C. (2018). Analysis of the ph-dependent fe (iii) ion chelating activity of anthocyanin extracted from black soybean [glycine max (l.) merr.] coats. J. Agric. Food Chem..

[161] Buchweitz M., Brauch J., Carle R., Kammerer D. (2013). Application of ferric anthocyanin chelates as natural blue food colorants in polysaccharide and gelatin based gels. Food Res. Int..

[162] Serobatse K.R., Kabanda M.M. (2016). Antioxidant and antimalarial properties of butein and homobutein based on their ability to chelate iron (ii and iii) cations: A dft study in vacuo and in solution. Eur. Food Res. Technol..

